# Effects of Mean Luminance Changes on Human Contrast Perception: Contrast Dependence, Time-Course and Spatial Specificity

**DOI:** 10.1371/journal.pone.0017200

**Published:** 2011-02-15

**Authors:** Markku Kilpeläinen, Lauri Nurminen, Kristian Donner

**Affiliations:** 1 Department of Behavioural Sciences, University of Helsinki, Helsinki, Finland; 2 Brain Research Unit, Low Temperature Laboratory, Aalto University School of Science and Technology, Espoo, Finland; 3 Department of Biosciences, University of Helsinki, Helsinki, Finland; Dalhousie University, Canada

## Abstract

**Background:**

When we are viewing natural scenes, every saccade abruptly changes both the mean luminance and the contrast structure falling on any given retinal location. Thus it would be useful if the two were independently encoded by the visual system, even when they change simultaneously. Recordings from single neurons in the cat visual system have suggested that contrast information may be quite independently represented in neural responses to simultaneous changes in contrast and luminance. Here we test to what extent this is true in human perception.

**Methodology/Principal Findings:**

Small contrast stimuli were presented together with a 7-fold upward or downward step of mean luminance (between 185 and 1295 Td, corresponding to 14 and 98 cd/m^2^), either simultaneously or with various delays (50–800 ms). The perceived contrast of the target under the different conditions was measured with an adaptive staircase method. Over the contrast range 0.1–0.45, mainly subtractive attenuation was found. Perceived contrast decreased by 0.052±0.021 (N = 3) when target onset was simultaneous with the luminance increase. The attenuation subsided within 400 ms, and even faster after luminance decreases, where the effect was also smaller. The main results were robust against differences in target types and the size of the field over which luminance changed.

**Conclusions/Significance:**

Perceived contrast is attenuated mainly by a subtractive term when coincident with a luminance change. The effect is of ecologically relevant magnitude and duration; in other words, strict contrast constancy must often fail during normal human visual behaviour. Still, the relative robustness of the contrast signal is remarkable in view of the limited dynamic response range of retinal cones. We propose a conceptual model for how early retinal signalling may allow this.

## Introduction

During the viewing of a natural visual environment, every saccade abruptly changes both the mean luminance and the contrast structure falling on the fovea or any other region on the retina. These changes are substantial [1], [2] and lead to neural responses which closely resemble responses caused by actual changes in the visual environment [3], [4].

Statistical analyses of natural images [5] suggest that mean luminance and local contrast at different retinal locations are only modestly correlated [1], [2], [ but see, 6]. This has led researchers to look for an analogous independence in the visual system. Reports of single cell recordings in the cat visual system suggest that the two stimulus parameters are quite independently represented in neural responses [2], [7].

While there is little doubt that “contrast constancy” may be regarded as one central function of the various known adaptation mechanisms of the visual system [8], [ however, see also 9], the completeness of success especially in a highly dynamic situation is debatable. The sensitivity adjustment of retinal neurons is fast but not instantaneous [10], [11], [12], [13], and the contrast response is inevitably mixed with the response to the luminance change itself [14]. Thus, immediately after a change in mean luminance, the contrast information mediated by the retina is unavoidably compromised. This is probably why psychophysical sensitivity to small luminance deflections is reduced when accompanied by a change in overall luminance level [15], [16], [17]. Even a short duration effect is highly relevant, as the next eye movement, and the consequent change in contrast and mean luminance, follows in some 200–300 ms [18].

Mean luminance and local contrast are the most fundamental features of the visual stimulus falling on any given part of the retina. It is essential to understand possible interactions in the processing of the two, as these are liable to affect many (if not all) aspects of visual performance. In the present work, we studied the effect of abrupt mean luminance changes on foveal contrast perception psychophysically in human subjects. The target stimuli were sine wave gratings or increment/decrement bars. To probe the time course of the effect of the luminance change, the target stimuli were presented at various delays in relation to the luminance step. We find that both upward and downward luminance steps attenuate the perceived contrast of a simultaneously presented target, the upward step somewhat more than the downward step. The effect was mainly subtractive and subsided in approximately 400 ms. We suggest that the failure of contrast-luminance independence under simultaneous changes of both parameters reflects the fact that luminance adaptation in the retina cannot be instantaneous.

## Results

### Experiment 1: The effect of a background luminance change on perceived contrast and detection thresholds

**Figure 1:**
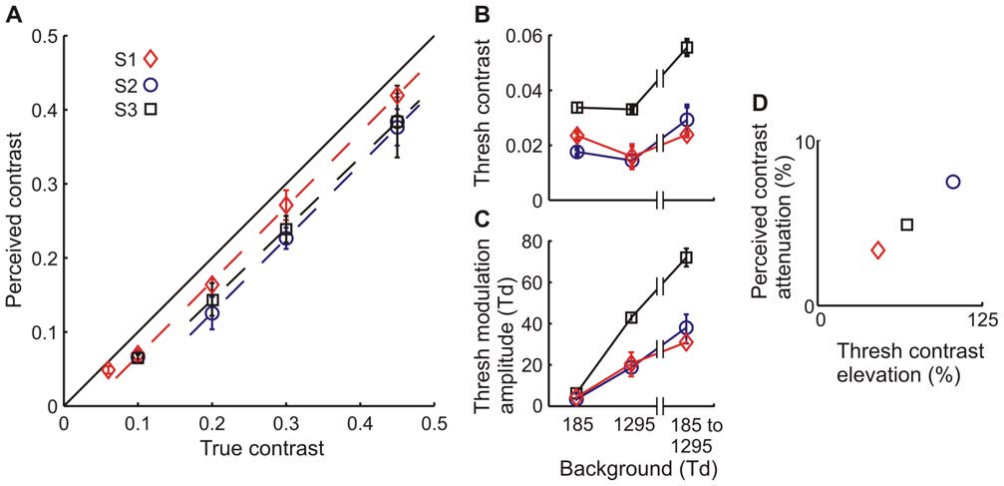
A simultaneous change in mean luminance attenuates contrast perception at threshold and supra-threshold contrasts. A) Perceived contrast of a sine-wave grating as a function of physical contrast. Mean luminance changed from 185 to 1295 Td. The solid 45 deg line indicates veridical contrast perception. The dashed lines are linear fits to the underlying data points for the three subjects. B) Threshold contrast, with steady mean luminance (185 Td and 1295 Td) and with the luminance step from 185 to 1295 Td. C) As in B, but with threshold values expressed as absolute modulation amplitudes. Error bars represent (here and henceforth) standard deviation. D) The threshold elevation and attenuation of perceived contrast vary very similarly between subjects.

**Figure 2:**
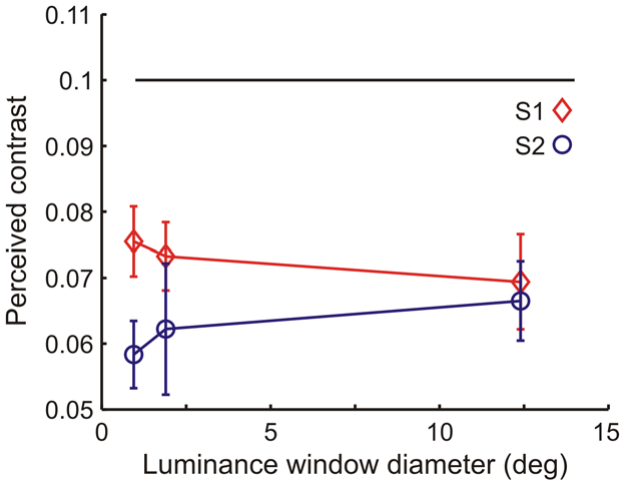
The window size of the luminance change has little effect on the strength of attenuation. The perceived contrast of a grating stimulus at contrast 0.1 as a function of luminance window diameter. Background luminance was stepped from 185 to 1295 Td simultaneously with target presentation. The largest luminance window diameter refers to the vertical size of the screen. The black horizontal line indicates the physical contrast of the target, i.e., a veridical match.

In experiment 1, the mean luminance of the entire screen increased by seven-fold (from 14 to 98 cd/m^2^, i.e., from 185 to 1295 Td with 4.1 mm pupil diameter) simultaneously with the onset of the target grating. The effect was always an attenuation of perceived contrast (see Fig 1). For target contrasts close to threshold, the attenuation was partly divisive, but upwards from contrasts 0.1–0.2 mostly subtractive. In other words, the perceived contrast decreased by a fixed amount for all target contrasts, except very close to detection threshold. For subject S1 (red diamonds) the attenuation was completely subtractive for contrasts 0.1 and higher. For subject S2 (blue circles) the attenuation was stronger overall, and completely subtractive for contrasts 0.2 and higher. For subject S3 (black squares) the attenuation was predominantly subtractive for contrasts 0.2 and higher. The slopes of the linear fits in Figure 2 differ negligibly from unity, being 0.995, 0.997, and 1.040 for subjects S1, S2, and S3, respectively. This indicates that a possible divisive component of attenuation is insignificant in the ranges fitted (from 0.1 upwards for S1 and from 0.2 for the two other subjects). The subtractive components have values 0.033, 0.075 and 0.049. As the staircase steps in this experiment were fixed fractions of the respective target contrast (i.e., relative to contrast), the subtractive attenuation observed cannot be the trivial consequence of a constant response bias. Moreover, in preliminary sessions without luminance steps, the contrast matches of all subjects were practically veridical, indicating negligible response bias at least in that situation (see Methods).

It is interesting to compare the attenuation strengths in individual subjects to their absolute detection thresholds for the same stimulus (Fig. 1B and C). Inter-subject differences in attenuation strength appear not to be related to *steady-state* detection thresholds. Instead, attenuation strength and threshold *elevation* covary. The luminance step has the smallest effect on both measures in Subject 1 and the greatest effect in subject 2. Figure 1D illustrates the strong relationship between threshold elevation and attenuation of perceived contrast. The threshold contrasts of subject 3 are clearly the highest in all conditions, possibly because of his shorter experience in psychophysical measurements.

### Experiment 2. Local or global origin of the attenuation effect?

In experiment 2, we studied the spatial properties of the attenuation effect by varying the size of the window within which the luminance change occurred. These experiments were carried out at target contrast 0.1, which had yielded the smallest inter-subject variation and largest fractional effect in experiment 1. Figure 2 presents perceived target contrast measured in two subjects with luminance steps occurring over windows of three different sizes, ranging from ca. 1 deg to full-field. The data show that, over this range, any possible effect of window size on attenuation magnitude is small. Thus we felt justified to use full-field luminance change in all other experiments, although some lateral spread of the attenuating signal especially over short distances cannot be definitely excluded.

### Experiment 3: Time course of attenuation

The purpose of experiment 3 was to measure the persistence of the attenuation effect. The time course was probed by presenting the target stimuli (contrast 0.1) with various delays relative to the luminance transition. Both upward (185 to 1295 Td) and downward (1295 to 185 Td) luminance steps were applied. In addition, to find out whether the change in perceived contrast was predominantly mediated by the peak or trough parts of the grating, experiments were done also with purely positive or negative contrast stimuli (increment or decrement bars).

**Figure 3:**
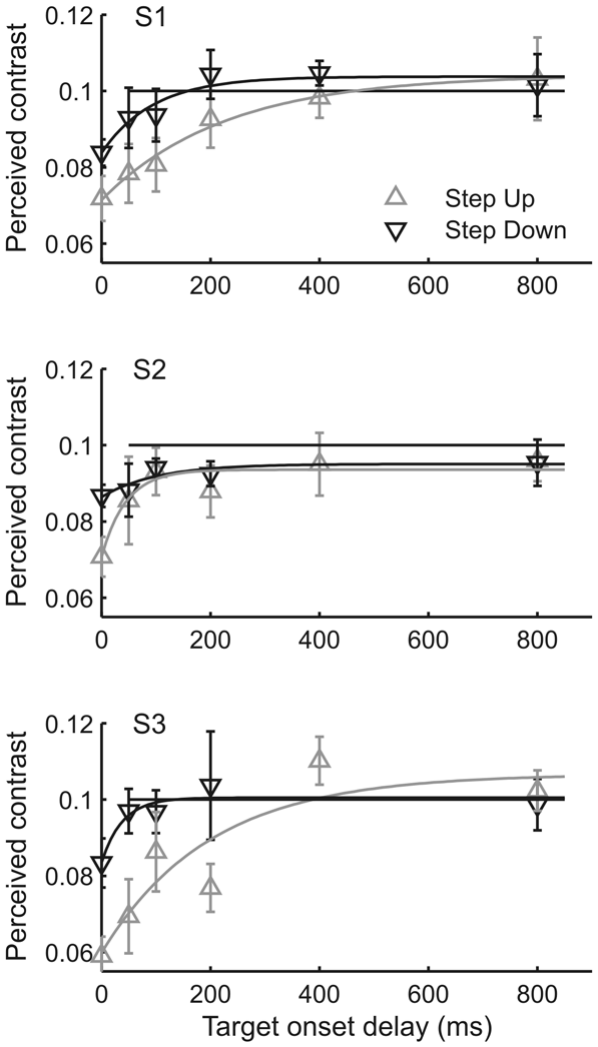
The attenuation of perceived contrast decays with increasing delay between luminance step and target onset. Mean luminance increased from 185 to 1295 Td (grey upward triangles) or decreased from 1295 to 185 Td (black downward triangles). The black horizontal line indicates the physical contrast of the sine-wave grating target, i.e., a veridical match. Smooth curves are best fitting exponential functions.

Attenuation was stronger with upward than downward luminance steps (Fig 3). In both cases the strongest effect was observed when the target was presented simultaneously with the luminance step. With the upward step in mean luminance (grey upward triangles in Fig 3), the target needed to be presented with approximately 400 ms delay in order to be perceived without significant attenuation. With the downward step (black downward triangles in Fig 3), the percept was veridical already after a 200 ms delay. The strength and time course of the attenuation effect was broadly similar for the different stimulus types (bars and gratings).

To quantify the decay of attenuation, we fitted to the data sets exponential functions of the form a - b× exp(-t/τ), where t is the delay (ms) of target onset relative to luminance transition, τ is the time constant and a and b are scaling parameters. The function should be regarded as an empirical description only, without theoretical significance. Table 1 presents the parameters for the various fits.

**Table 1:** Parameters for the fits in figures 3 and 4.

		Grating		Increment bar		Decrement bar	
		Step	Step	Step	Step	Step	Step
Subject	Param	Up	Down	Up	Down	Up	Down
	a	0.1042	0.1038	0.0986	0.1038	0.1018	0.1056
S1	b	0.0328	0.0200	0.0437	0.0258	0.0387	0.0254
	τ	227	95	231	42	228	144
	a	0.0940	0.0951	0.0742	0.0986	0.0743	0.1002
S2	b	0.0228	0.0087	0.0248	0.0287	0.0246	0.0268
	τ	45	101	153	52	108	54
	a	0.1067	0.1005				
S3	b	0.0468	0.0170				
	τ	199	38				

**Figure 4:**
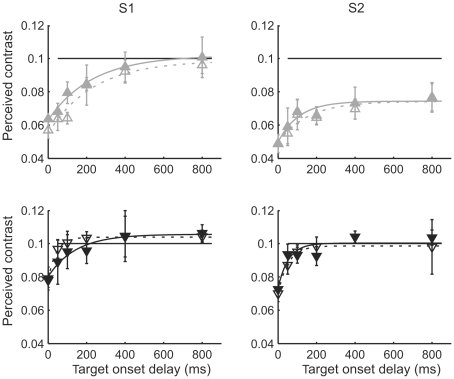
Effects on increment and decrement bars are consistent with effects on gratings. Mean luminance increased from 185 to 1295 Td (grey upward triangles) or decreased from 1295 to 185 Td (black downward triangles). The bar was a decrement (filled markers) or an increment (open markers) relative to the background luminance. The black horizontal line indicates the physical contrast of the target, i.e., a veridical match. Smooth curves are best fitting exponential functions.

Results of the experiments with either increment or decrement bars as contrast stimuli, i.e., with a single contrast polarity relative to the mean level, are shown in Figure 4. Regardless of contrast polarity, attenuation was always stronger and more persistent with the upward step (grey upward triangles) than with the downward step (black downward triangles). The time constants describing the decay of the effect were also consistently larger in the step-up condition. Finally, there appeared to be a general trend that for a given step direction, attenuation was more persistent for the stimulus type which modulates to the same direction as the mean luminance, i.e., for the increment bars when presented with an upward luminance step and for the decrement bars when presented with a downward step. The increment/decrement data in the step up condition for subject 2 should be regarded with some caution, as the contrast percept did not return to baseline (veridical) even after 800 ms.

## Discussion

### An abrupt change in mean luminance attenuates perceived contrast

**Figure 5:**
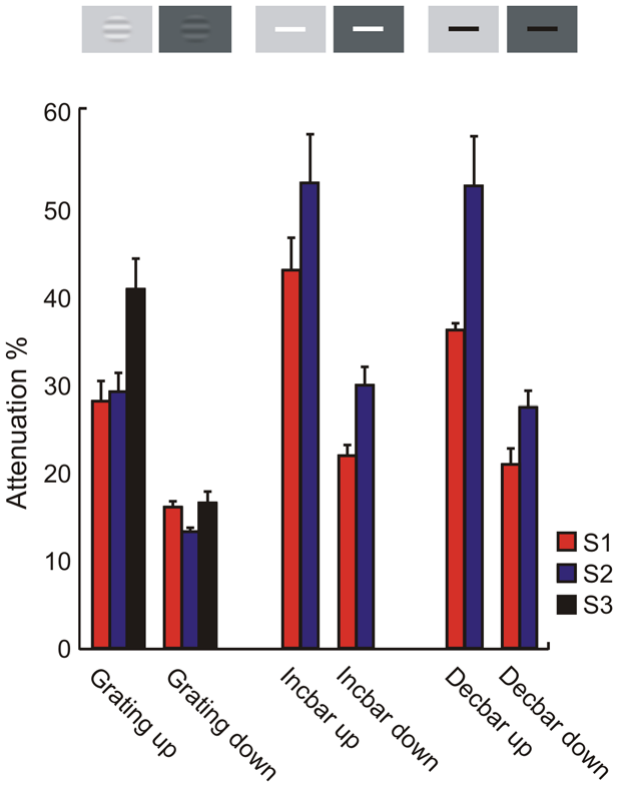
The luminance step caused significant attenuation of perceived contrast, regardless of target stimulus type. Summary of attenuation strengths (%) at 0 ms delay, 0.1 target contrast and a full field luminance change for all target stimulus types. Decbar refers to decrement bar, incbar to increment bar.

A moderate step (0.85 log units) in mean luminance in an ecologically relevant [1], photopic luminance range was found to attenuate the perceived contrast of a simultaneously presented target for a wide range of supra-threshold contrasts and different target types (see Fig 5). With an upward luminance step, the attenuation effect was almost purely subtractive in the contrast range from 0.1 or 0.2 up to 0.45. The strength of the effect did not depend significantly on the spatial extent of the field over which luminance changed, and it subsided in approximately 400 ms. A downward luminance step had a somewhat smaller and less persistent effect. Similar direction asymmetry for the persistence of desensitization has earlier been observed in psychophysical experiments measuring *thresholds* for detecting small luminance increments [17].

### Asymmetry of upward and downward luminance steps

When trying to understand how the various effects may arise, the basic situation in our measurements should be clearly kept in mind. The subject compared the contrast of the target, *all parts of which* were either increments (in the step-up condition) or decrements (in the step-down condition) relative to the original adaptation level, with the contrast of the comparison stimulus, the luminance of which modulated around the new adaptation level. At least two retinal factors, which are not mutually exclusive, may contribute to the asymmetry in the effects of upward and downward luminance steps. Firstly, an upward step will transiently compress and a downward step will expand the dynamic response range in retinal cone photoreceptors [19]. The range compression is liable to decrease the response to the large target modulation in the step-up condition, while the expansion of response range associated with a step-down could partly counteract attenuation caused by other possible mechanisms. Secondly, there is a functional asymmetry of the ON and OFF channels of the visual system. ON ganglion cells have a larger response range for decrements, than OFF cells have for increments. OFF cells are, in fact, largely unable to respond to increments [20], [21]. This asymmetry has been suggested to underlie [21] the higher psychophysical sensitivity to luminance decrements in comparison to increments [22], [23].

### Subtractive attenuation

For a wide range of contrasts (from 0.1 or 0.2 up to at least 0.45), an upward luminance step had an almost purely subtractive effect on perceived contrast (Fig. 1). This result was quite surprising, as earlier psychophysical measurements of *thresholds* have suggested a central role for divisive attenuation [16], [24]. Divisive attenuation would indeed be the type of effect primarily expected from the compressive stimulus-response function of retinal cones. Based on data from macaque cones, the response to a step from 185 Td to 1295 Td can be estimated to use up about 60% of the cone dynamic range at peak, thus compressing the response range available for simultaneous contrast stimuli to roughly 40% of the original (see Fig 7 in [19]). The simplistic prediction is that all contrast signals would be divided by the factor 2.5, implying substantial divisive attenuation. There are of course many possible accessory mechanisms (in transmission or e.g. adaptation) and assumptions by which the photoreceptor nonlinearity can be reconciled with the subtractive and (by comparison) moderate suppression observed in this study.

There is, however, an alternative line of thinking about the mechanistic path from photoreceptor responses to perception, a model that has previously been successfully used to explain data on ganglion cell responses and psychophysical brightness perception. [25], [26], [27], [28]. According to this model, the signal is invulnerable to the above described saturation of photoreceptor responses by virtue of two realistic key assumptions. Firstly, the model assumes that the primary contrast metric in responses to stimuli well above the detection threshold is the steepness of the *early rise* of photoreceptor responses, when the response has reached a small criterion amplitude [26]. The initial rise of photoreceptor responses is known to depend *linearly* on stimulus intensity [29], [30]. Such linearity implies that:
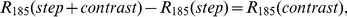
(1)where (and henceforth) *R_185_* is the relevant response of cones when adapted to the lower (185 Td) luminance and *R_1295_* when adapted to the higher (1295 Td) luminance. Secondly, it is assumed that the subtraction in eqn. (1) is implemented at an early stage in the retina (e.g. by horizontal cells that may compute average luminance over large receptive fields), and that the uncompromised contrast signal can thus be delivered to the read-out mechanism (e.g., the ganglion cells). Now, the early rise of photoreceptor responses has been shown to be approximately invariant against a wide range of steady adapting luminances [31], [32], [33], [34], implying that:
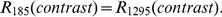
(2)


Substitution into equation (1) yields:
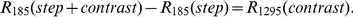
(3)


To summarize, the contrast signals produced by the target stimulus (the left side of equation 3), and the comparison stimulus (the right side of equation 3), should be equal. The straightforward interpretation would be that the contrast of the target should be perceived veridically, i.e., with no attenuation, even when presented simultaneously with the luminance step. Again, it would be easy to think of accessory mechanisms and assumptions to reconcile this framework with the moderate subtractive suppression observed in the data.

However, since the first hypothesis, based on response amplitudes compressed by the saturation of photoreceptors, predicts divisive attenuation that is too strong especially at higher contrasts, and the latter hypothesis, based on the linear early rise of photoreceptor responses, predicts no attenuation, a physiologically justified combination of the two seems an interesting and quite plausible possibility. We suggest that the signalling of supra-threshold contrast basically depends on the early response rise, but that it fails to follow the strictly linear idealization of equations 1–3, because the read-out mechanism must integrate the subtraction signal over a finite time, until some criterion (e.g., in terms of signal-to-noise ratio) is achieved. At low contrasts, long stretches of the later, “compressed” parts of cone responses have to be included before such a criterion is reached, and this causes a deviation from the veridical match that would follow from strict linearity. The higher the contrast, however, the earlier the subtraction signal reaches the criterion, and the less of the “compressed” response segments will have to be included in the integral. To summarize, if the relative contribution from compressed response segments decreases with increasing contrast, attenuation as function of contrast may appear as approximately subtractive rather than divisive.

### The time-course of the attenuation effect

The experiments where the target was presented with different delays relative to the luminance step show that the attenuation decays quite rapidly, reaching negligible levels in approximately 200–400 ms (see Figs 3 and 4). This is in line with earlier psychophysical findings with threshold level stimuli [12], [15], [17]. By definition, it indicates adaptation of some neural stage(s), but the current data does not allow a precise localization. We note, however, that the observed time course is roughly consistent with the time scale of “fast” adaptation in cones, which restores a large part of the light-sensitive current initially turned off at the peak of the response to a step of light[19]. It is also worth noting that our adapting luminances (185 to 1295 Td) fall mainly in the range where the primary adaptation site at least in monkey retina is at the receptoral rather than post-receptoral level [35]. It needs to be noted, though, that in the experiment with increment/decrement bar stimuli and upward luminance step (grey triangles in Fig 4), the data of subject 2 show that in him only part of the initial (and exceptionally large) attenuation could be reversed by fast adaptation.

The approach of experiment 3, where the adaptation state of the target was varied by varying the onset time of the target, while keeping the onset time of the comparison constant, inevitably caused the interval between the target and comparison stimulus (the ISI) to vary as well. While a confounding effect of ISI would be possible in principle, we find it highly unlikely for the following reasons: Most of the attenuation decay occurred in the first 200 ms of target delay and very little was observed at longer delays (from 400 to 800 ms). In terms of ISI, this would mean that, curiously, a change from 1000 to 1400 ms is ineffective, but a change from 1600 to 1800 ms has a large effect. Moreover, experiments on simple contrast matching have shown no effect of changing ISI between 1000 and 3000 ms [36].

Despite the clear effect that a luminance step has on contrast responses, both at the retinal and at the perceptual level, it might be thought that the gain control systems of the visual system work well enough, after all, and that the effects observed here are too small and short-lived to be relevant. However, during natural viewing where saccades and fixational eye movements change the stimulus situation at all points of the retina at least every 200–300 ms [18], [37], the attenuation effect observed here is likely to cause some aspects of a natural image to go unperceived. This could occur not only because the fragment would remain below detection threshold, but also because another fragment with the same contrast, but affected by a smaller mean luminance step, would be more salient and prevail during the next fixation reallocation. In addition, the reliability of the neural or perceptual representations of stimuli are likely to be affected as the early part of neural responses, which is generally considered the most reliable part [38], [39], [40], is especially vulnerable to the effect of a simultaneous change of luminance and contrast. Finally, to offer some perspective, the magnitude and duration of the observed effects are comparable to those found for cross-orientation overlay masking [41] and iso-orientation surround suppression [42]. These effects are generally considered to be perceptually relevant.

### The attenuation effect is local

In most experiments of the present study, like in most of the earlier studies, mean luminance was changed over a considerably larger area than covered by the target stimulus. This raises the question whether the observed effect is local or contextual. Webb et al.[43] showed that a uniform flickering surround field can suppress the contrast responses of a neuron in macaque V1. We addressed this question by an experiment where the luminance change was restricted to smaller windows, finding that window size had little or no effect on attenuation strength (Fig 2). However, even the smallest luminance window was somewhat larger than the target stimulus and we cannot rule out that some surround suppression was present. Nevertheless, the result indicates that the attenuation mainly reflects stimulus-response properties of neurons covered by the target rather than suppression from a wider surround and that the adaptation to the new mean luminance between the luminance step and the onset of the comparison stimulus (1800 ms later) occurs quite locally indeed. The (relatively small) difference between subjects 1 and 2 observed with the smallest window size, if not random, may suggest the presence of some second-order surround effects, e.g., differences in a Westheimer-type [44] balance of inhibition and disinhibition in the near surround.

### What happens between the eye and the motor response?

Two recent single-cell studies from cat visual system have emphasized luminance-contrast independence, which is supposed to “match” the independence found in natural scenes. These reports suggest that a transition in mean luminance has little if any effect on the strength of contrast responses [2], [7]. It would be very surprising if an effect that is present both in the input (the retina) and in the output (behavioral responses) would be absent at intermediate stages. Our present results allow us to rule out two potential explanations of the apparent discrepancy, based on differences in the stimulus situation: (1) The earlier psychophysical studies measured thresholds, while the single-cell studies [2], [7] used mostly supra-threshold stimuli and did not analyze possible threshold elevation. As shown in our Figure 1, however, a change in mean luminance *both* elevates detection thresholds *and* attenuates perceived supra-threshold contrast. (2) The earlier psychophysical studies have mostly used pure contrast increments or decrements whereas the single-cell studies used stimuli modulated in both directions around the mean luminance. We find, however, that a change in mean luminance has very similar effects on the perception of both stimulus types (Fig 5).

Probably, the apparent discrepancy between the cited psychophysical and electrophysiological studies depends on differences in their analytical focus. Whereas the psychophysical studies (including ours) have been interested in the transient desensitization caused by a luminance change of a certain size, the single-cell studies looked for contrast-luminance independence in responses averaged over different magnitudes and directions of luminance change, and over the time of drift of a contrast grating. In either case, specific effects of the luminance transition on the early part of the responses have probably been blurred by averaging or simply not considered to be of great interest. Indeed, one can observe in Figure 4 of Geisler et al. [7] that the initial parts of the contrast responses are inversely related to the magnitude of the luminance change rather than luminance per se.

### The visual environment and the visual system: a perfect match?

Numerous studies have recently demonstrated that the visual system is designed to match the statistics of natural images [1], [2], [5], [45]. Of particular relevance to the current study are those concerning local luminance and contrast [1], [2]. These studies suggest that the mammalian visual system reproduces the contrast-luminance independence of natural images with high precision. Due to the limited operating range of visual neurons, such performance requires extremely efficient regulation mechanisms. Several studies show that the visual system meets these requirements to a remarkable extent [see 8, for a review]. However, adaptation of a visual neuron to a certain stimulus parameter, here a (changed) level of mean luminance, cannot be instantaneous, as it necessarily requires sampling over time to determine the (new) value of that parameter. Further, the response of the neuron to the luminance change itself will compromise contrast transfer for a certain period after the change, regardless of the speed of adaptation in the strict sense of gain adjustment.

In conclusion, our data show that conscious perception of various contrast stimuli is significantly affected by co-occurring changes in mean luminance. The effect was fairly similar for sinusoidal contrast gratings and for bars representing pure increment or decrement contrasts. Attenuation was found to be transient, subsiding within 200–400 ms from the luminance step, but persistent enough to be of relevance to vision, considering the rapid refresh rate of the retinal image during normal viewing of natural scenes. The attenuation may be largely due to the limited response range and finite adaptation speed of retinal cones. This would imply that, when luminance and contrast change simultaneously, as they do with saccades, the two cannot be strictly separable in neural responses at any level of the visual system.

## Materials and Methods

### Participants

Altogether three subjects (all male, age 24–33 years) participated in the study. Subjects 1 and 2 were authors of the paper and subject 3 was a university student, naïve to the purposes of the study. He received a small monetary compensation. All subjects had normal, uncorrected vision.

### Ethics statement

The participants gave written informed consent. The study was approved by the ethical committee of the faculty of behavioural sciences in the University of Helsinki.

### Apparatus

Stimuli were created with Matlab 7 (MathWorks Inc, Natick, MA, USA), controlled with a Visage (Cambridge research systems, Rochester, UK) frame buffer, and displayed on a VisionWorks (Vision Research Graphics, Durham, NH, USA) monochrome display with a fast p46 phosphor. The spatial resolution of the display was 800×600 (300 mm×225 mm) and the temporal resolution 160 Hz. The conventional gamma correction was carried out with a ColorCal luminance meter and Cambridge research systems calibration routines. Two additional corrections were required. Firstly, in monochrome displays, each point's luminance is somewhat dependent on the mean luminance of the screen. Thus, we calibrated the monitor separately for different mean luminance levels. Secondly, the display's mean luminance drifted slightly downwards during a measurement when high mean luminance levels were used. Trial-by-trial LUT updates were used to compensate for the drift. In addition, the monitor remained without input for a sufficient period after every measurement so that it returned to its original luminance range. Since abrupt and extensive luminance changes were instrumental in this study, a Thorlabs FDS100 (Thorlabs Inc., Newton, NJ, USA) photodiode and a Tektronix TDS3012 (Tektronix Inc., Beaverton, OR, USA) oscilloscope were used to determine that the luminance transitions occurred fully within one refresh interval. Viewing distance was always 103 cm. The subjects viewed the stimuli monocularly. Retinal illumination was controlled with an artificial pupil with an effective corneal diameter of 4.1 mm. The natural pupil was fully dilated. The other eye was occluded.

### Stimuli

In experiment 1, the stimuli were circular patches of sine wave grating. The edges of the grating windows were smoothed with half a cycle of raised cosine function (plateau diameter 0.5 deg, edge width 0.1875 deg). Michelson contrast of the target stimulus was varied (0.1–0.45) between measurements. The spatial frequency of the gratings was 4 cycles per degree of visual angle. Grating orientation was horizontal. The target grating was always presented simultaneously with an upward change in mean luminance of the entire screen (from 185 to 1295 Td, from 14 to 98 cd/m^2^). In the measurement of detection thresholds, target contrast varied during the course of the measurement. Both upward (from 185 to 1295 Td) and downward (from 1295 to 185 Td) changes in mean luminance were used. For comparison, data were also collected with steady mean luminance levels (1295 or 185 Td). The illumination levels used in the experiment fall safely within the range within which conventional steady state contrast constancy holds [46], [47].

In experiment 2, the stimuli were gratings like in experiment 1, presented simultaneously with the change in mean luminance. The change was carried out in three different spatial windows: the entire screen, a raised cosine window with plateau 1.9 deg and edge width 0.375 deg and a raised cosine window with plateau 0.95 deg and edge width 0.375 deg. The smallest window size was such that fixational eye movements would not disrupt the luminance adaptation process between target presentation and comparison stimulus presentation [48].

In experiment 3, the stimuli were either gratings as in experiment 1 or bars of increment or decrement luminance with height of 1/8 deg and width of 1 deg (i.e., approximately the spatial dimensions of a central half cycle of the grating stimulus). Contrast (modulation amplitude divided by background, equal to Michelson contrast for gratings) was always 0.1 and the target stimulus was presented at various delays (0–800 ms) relative to the mean luminance transition. Both upward (from 185 to 1295 Td) and downward (from 1295 to 185 Td) changes in mean luminance were used.

### Procedure

**Figure 6:**
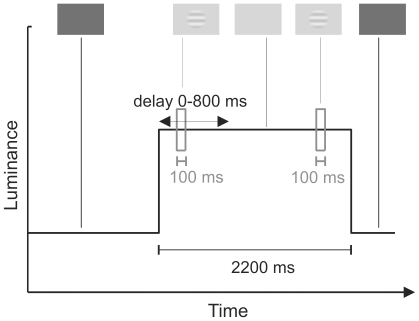
The time course of a typical stimulus trial. In the case illustrated here, mean luminance changes upward and a target grating is presented with a delay of 200 ms relative to the luminance change.

The time-course of a single trial is presented in Figure 6. In the beginning of each measurement, the subject adapted to the baseline luminance of the measurement for a minimum of 40 seconds. During the measurement, each trial proceeded as follows: First, a fixation stimulus (a 2×2 pixel central square and a 1-pixel-wide circle with a diameter of 1.6 deg, luminance 80% of background, duration 200 ms) and a subsequent blank period (300 ms) were presented twice. Then, the mean luminance of the screen was changed abruptly. The target stimulus (duration 100 ms) was presented with either a simultaneous or a delayed onset relative to the change in mean luminance. A comparison stimulus (100 ms) was presented 1800 ms after the luminance change. The mean luminance of the screen returned to the baseline level 300 ms after the comparison stimulus disappeared. The baseline luminance was then shown for 8 seconds before the next trial. In conditions where there would have otherwise been a period longer than 500 ms between the last presentation of the fixation stimulus-blank period combination and the onset of either the target or the comparison stimulus, the combination was presented again during that period. The multiple presentations of the fixation stimulus during the trials served to ensure that perception remained with the stimulated eye. Between measurements the subject viewed another display with approximately the same luminance and color as was the baseline in the next measurement. There were no other light sources in the room.

The presentation time of the comparison stimulus was determined in preliminary experiments in which a target and a comparison were presented 1000 ms apart at various delays after the luminance change. Those experiments suggested that matching becomes veridical, i.e., effects of the luminance change vanish, well before 1800 ms. The data in our experiment 3 support this. However, even if there is some effect left, it does not compromise our main conclusions, since all targets are always compared against comparison stimuli presented with the same delay (i.e., against the same “gold standard”).

The perceived contrast of the target stimulus was measured with a 2-interval forced-choice staircase procedure. The subject's task was to indicate with a key stroke, whether the target or the comparison stimulus appeared to have a higher contrast. If the subject judged the comparison stimulus to have a lower contrast than the target, the contrast of the comparison stimulus was increased. If the subject judged the comparison stimulus to have the higher contrast, its contrast was decreased. A reversal point of a staircase is a point where the direction of the adjustment of the contrast of the comparison stimulus changes. One measurement included two randomly interleaved, independently progressing staircases, each containing 8 reversal points. The result of each staircase was the mean of the last 4 reversal points from each staircase. The first 4 reversal points of each staircase were considered practice and omitted from the calculation. The individual data points (and accompanying error bars) reported in this paper are the means (and standard deviations) of at least 4 staircases. In the beginning of each measurement session, the subjects always performed a few measurements with constant mean luminance (i.e., matching target and comparison stimulus without luminance step) to check that they achieved a veridical match and thus had no significant response bias related to the order of presentation [49].The mean perceived contrasts in the practice runs (always done with target contrast 0.1) were 0.103±0.0006 for S1, 0.102±0.0001 for S2 and 0.101±0.003 for S3, respectively.

In the measurement of detection thresholds in experiment 1, an unbiased 1-interval method presented by Kaernbach [50] was used. Simultaneously with the change in mean luminance, a target grating was presented with 50% probability. To reduce uncertainty [51], a circle (the same as in the fixation stimulus) was presented around the target [see, 52]. The subject's task was to indicate, whether the target had been presented or not. In each measurement there was one staircase with 10 reversal points. Detection threshold contrast corresponding to 83.5% correct was calculated as the mean of last 8 reversal points. As there was no comparison stimulus, mean luminance returned to the baseline level 500 ms after target presentation.
